# The effective interplay of (non-) selective NSAIDs with neostigmine in animal models of analgesia and inflammation

**DOI:** 10.1186/s40360-021-00488-9

**Published:** 2021-05-01

**Authors:** Mennatallah A Gowayed, Amany Abdel-Bary, Rasha A El-Tahan

**Affiliations:** 1 Department of Pharmacology & Therapeutics, Faculty of Pharmacy, Pharos University in Alexandria, Alexandria, Egypt; 2 Department of Pathology, Faculty of Medicine, Alexandria University, Alexandria, Egypt; 3 Department of Biochemistry, Medical Research Institute, Alexandria University, Alexandria, Egypt

**Keywords:** Neostigmine, Celecoxib, Diclofenac, Writhing, formalin induced edema

## Abstract

**Background:**

Surgical procedures cause perioperative immunosuppression and neuroendocrine stress, exerted by activation of the autonomic nervous system and the hypothalamic-pituitary-adrenal axis. The acetylcholinesterase inhibitor (ACHEI); neostigmine, is known clinically for its analgesic effect in the perioperative phases proving high efficacy; besides possessing anti-inflammatory properties controlling immune cells and cytokine level. Hence, this study evaluated and compared the analgesic and anti-inflammatory activities of the combination of selective Cox-2 inhibitor; celecoxib, with neostigmine *versus* a combination of the non-selective Cox inhibitor; diclofenac, with neostigmine; in different experimental models of analgesia and inflammation in rats.

**Methods:**

Analgesic activity of neostigmine with/without diclofenac or celecoxib was assessed in female Sprague-Dawely rats using the tail clip model and acetic acid induced writhing. Serum level of β-endorphin was assessed after the tail clip test. The anti-inflammatory activity was evaluated using acute and sub-chronic formalin induced paw edema. At the end of the sub-chronic formalin test, blood samples were collected for analysis of anti-inflammatory, liver and kidney function markers. Livers, kidneys and hind paws were also examined histopathologically.

**Results:**

Addition of neostigmine to selective or non-selective NSAIDs (celecoxib or diclofenac) causes an increased level of analgesia of NSAIDs with rapid onset of action and short duration, while causing potentiation of the anti-inflammatory effect of neostigmine as seen in the tail clip, writhing, formalin test, Cox-1 and Cox-2 activities, serum β-endorphin, TNF-α, NF-кB and HS-CRP. All combinations of this study disturb some kidney and liver functions, however with normal histopathological appearances, while hind paws reveal improved inflammatory infiltration in all treated groups.

**Conclusions:**

Selective and non-selective NSAIDs examined in this study could be good adjunct options to general anesthetic agents and neostigmine in perioperative stages, an outcome that needs further clinical investigations.

**Supplementary Information:**

The online version contains supplementary material available at 10.1186/s40360-021-00488-9.

## Background

Non-steroidal anti-inflammatory drugs (NSAIDs) are known to provide only symptomatic relief of acute as well as chronic pain and inflammation. The known mechanism of NSAIDs is the inhibition of the enzyme cyclooxygenase (Cox), which consists of two isoforms known as Cox-1 and Cox-2 [1], each has a different pattern of expression and function. Cox-1 is constitutively expressed and is present in most tissues in high levels, whereas Cox-2 is inducible reaching very high levels during inflammation. Under physiological conditions, low levels of Cox-2 are usually detectable in some tissues [2].

It has been generally accepted that the inhibition of Cox mediates the anti-inflammatory, antipyretic and analgesic effects of NSAIDs [3], where analgesia has been associated with selective Cox-2 inhibition. However, the selective inhibition of Cox-2 seemed not to be enough to mediate the anti-nociceptive activity of NSAIDs in several models of acute pain [4]. Moreover, the long term use of these agents and inhibition of Cox-1 and Cox-2 has been associated with serious side effects, particularly gastrointestinal and cardiovascular [5]. Hence, newer regimens are needed to reduce the risks of NSAIDs while achieving better analgesic and anti-inflammatory effects.

The role of the cholinergic system in nociception has been recognized in the past few years, nominating acetylcholine as an endogenous anti-nociceptive compound [6–8]. On the other hand, the cholinergic anti-inflammatory pathway has been elaborating in treatment of neuro-inflammation and arthritic diseases [9–11]. Thus, acetylcholinesterase inhibitors (ACHEIs) such as neostigmine (Neo) have shown to produce analgesia after systemic and intrathecal administration during anesthesia [12, 13], besides possessing anti-inflammatory properties controlling immune cells and cytokine level. This positive modulatory action of cholinergic agonists enhancing analgesia has been related to induction of opioid or clonidine pathways [14]. An increase in the endogenous opiates, beta endorphins (β-endorphins), is known to occur in the brain and spinal cord in response to physiologic stressors such as pain. Upon binding to their mu-opioid receptors in peripheral and central nervous system, they show analgesic effect due to elimination of pain sensation by reducing the extent of nociceptive action potential [15]. The degree of pain experienced in patient during and after surgery correlated positively with plasma β-endorphin level in earlier studies [16, 17]. Non-opioid medications were also found to affect plasma β-endorphin level, where the Cox-2 inhibitor, rofecoxib; decreased pain intensity in osteoarthritis patients. An effect that was associated with β-endorphins synthesis and durability [18]. Hence, measuring serum β-endorphin level will reflect the status of analgesia and pain sensation experienced in the current study.

A study of the characteristics of the interaction between cholinergic agent like neostigmine and NSAIDs has not been performed for all combinations of drugs. Thus, the aim of this study was to evaluate and compare the analgesic and anti-inflammatory activities of the combination of selective Cox-2 inhibitor; celecoxib; with the ACHEI; neostigmine *versus* a combination of the non-selective Cox inhibitor; diclofenac, with neostigmine; in different experimental models of analgesia and inflammation in rats.

## Methods

### Animals

Female Sprague-Dawley rats aged 6–7 weeks, weighing 150–180 g were used throughout the experimental work. Rats were obtained from the animal house of Pharos University in Alexandria, Egypt, and kept under observation for at least one week prior to study with free access to food and water. The Research Ethical Committee of the Medical Research Institute, Alexandria University, Egypt approved the study (No: AU0122021132). All procedures were performed according to ARRIVE guidelines and comply with the National Research Council’s guide for the care and use of laboratory animals. The study was conducted in accordance with the Basic & Clinical Pharmacology & Toxicology policy for experimental and clinical studies [19]. The number of animals was kept to a minimum, the duration of experiment was as short as possible and the animals were killed immediately after cessation of the experiment. For evaluation of the analgesic effect algesiometric tests (*tail clip and writhing tests)* were performed. For evaluating the anti-inflammatory effects, rats have undergone acute and sub-chronic formalin test. In the algesiometric and acute formalin tests, each animal was used once and received only one dose of the test drug. All observations were performed by two observers in a randomized and blind manner.

### Drugs

Acetic acid and formalin were purchased from El-Gomhouria for trading Pharmaceuticals, Chemicals and Medical Appliances, Alexandria, Egypt. Celecoxib capsules from Pfizer Pharmaceutical Company, Alexandria, Egypt, and neostigmine ampoules from Amriya Pharmaceutical industries, Alexandria, Egypt, and diclofenac sodium ampoules from NOVARTIS, Alexandria, Egypt were used in the experiment. All ELISA kits were purchased from MyBioSource, San Diego, USA. The activities of Cox-1 and Cox-2 were determined using colorimetric assay kit (Cayman Chemical Company; Ann Arbor, MI, USA).

### Experimental design

Rats were allocated into seven groups of 8 rats each as follows: Group I, normal rats receiving sterile saline, group II, positive control subjected either to tail clip or acetic acid or formalin and not treated, group III, receiving 0.2 mg/kg neostigmine [20], group IV, receiving 10 mg/kg diclofenac sodium [21], group V, receiving 2 mg/kg celecoxib [22], group VI, receiving a combination of neostigmine (0.2 mg/kg) and diclofenac sodium (10 mg/kg), group VII, receiving a combination of neostigmine (0.2 mg/kg) and celecoxib (2 mg/kg). All drugs were freshly prepared and dissolved in saline. Doses were chosen from previous studies and have been tried in a pilot phase before the start of the real experiment. Drugs were injected intraperitoneal 30 min once before performing the tail clip, writhing and acute formalin study. In the sub-chronic formalin test, drugs were given daily after induction of inflammation with formalin for 5 days (the first dose started 5 h after formalin injection). All procedures were performed rapidly with a high degree of accuracy and reproducibility. At the end of the study, rats were anesthetized using a mixture of 80 mg/kg ketamine and 5 mg/kg xylazine and euthanized using cervical dislocation. Samples were then collected for further biochemical and histological studies. Rats were then kept frozen till incineration.

### Assessment of analgesic activity

#### HAFFNER’s tail clip method

An artery clip was applied to the root of the tail of rat (approximately 1 cm from the body) to induce pain [23] and the reaction time was noted. The animal quickly responded to this noxious stimulus by biting the clip or the tail near the location of the clip. The time between stimulation onset and response was measured by a stopwatch in 1/10 seconds increments. In all groups, tail clip test was performed preceding drug administration, and at 15, 30, 45- and 60-minutes following drug administration. The test latency (reaction time) at each time interval was calculated. The % analgesia was calculated (% analgesia = MPE = (TL – BL / ML – BL) *100), where MPE = max possible effect, TL = test latency, BL = control latency or basal latency and ML = max latency or cut off time. Cut off time of ten seconds was obliged in all sets of experiments [24] .

#### Writhing test

Pain was induced by injection of irritant (0.7 % acetic acid) into the peritoneal cavity of rats [25]. The animals reacted with a characteristic abdominal cramp, which is called writhing.

Only rats that showed a positive response to acetic acid were used for the test on the next day. Test animals were administered the drug or the standard 30 min prior to acetic acid administration, then rats were placed individually into glass cage, observed for a period of fifteen min and the number of writhes was recorded for each rat. The % inhibition was calculated (% inhibition = (Wc –Wt) *100 / Wc), where Wc = number of writhes in control rats and Wt = number of writhes in test rats. Drugs showing less than 70 % inhibition were thought to have minimal analgesic activity [24].

#### Serum β-endorphin level

At the end of the tail clip test, after 60 min, rats were euthanized as mentioned earlier and blood was collected from aorta for measurement of the level of β-endorphin in all tested groups.

### Assessment of anti‐inflammatory activity

#### Acute anti‐inflammatory test

For screening of anti-inflammatory drugs, 0.05 ml of 5 % solution of formalin was injected subcutaneously into the plantar side of the left hind paw of rats. The tested drugs were administered intraperitoneally 30 min before the challenge with formalin, to test the ability of such agents to prevent the edema produced in the hind paw of the rat after injection of the phlogistic agent. The paw diameter was measured by vernier caliper before the start of the experiment, then again after 3 h, and eventually 24 h after the challenge [26]. The % inhibition at different time interval was calculated as % inhibition = (paw volume in control rats – paw volume in test rats) divided by paw volume in control group and multiplied by hundred [24].

#### Sub‐chronic anti‐inflammatory test

Sub-Chronic phase of inflammation was enabled by subcutaneous injection of 0.05 ml 5 % solution of formalin into the plantar side of the right hind paw of rats. Tested drugs were administered intraperitoneally 5 h after formalin injection and then daily for five days [26]. The paw diameter was measured before the start of the experiment and then daily by vernier caliper. The % anti-inflammatory effects of drugs was calculated as: % anti-inflammatory effect = (mean paw volume in control rats – mean paw volume in test rats) divided by mean paw volume in control rats and multiplied by hundred [24].

At the end of experiment rats were euthanized using a mixture of 80 mg/kg ketamine and 5 mg/kg xylazine. Blood was collected from the posterior vena cava through a laparotomy incision to determine some serum parameters using ELISA. The anti-inflammatory parameters tumor necrosis factor- ɑ (TNF-ɑ), high sensitivity C-reactive protein (HS-CRP) and nuclear Factor-кB (NF-кB) were measured. The Cox activities (Cox-1, Cox-2 and Total Cox) assays were determined according to the procedures used by Kargman et al. [27]. The Kit (Cayman Chemical Company; Ann Arbor, MI, USA) includes isozyme-specific inhibitors for distinguishing Cox-2 from Cox-1 activity. Protein was determined using a modified method of Lowry et al. [28]. The specific activity of each enzyme was determined by dividing its activity by the protein concentration in the sample (U/mg protein) [27]. Also, aspartate transaminase (AST) and alanine transaminase (ALT) as indicators of liver function were measured. Creatinine and urea were determined spectrophotometrically. Livers, inflamed hind paws and one kidney were removed, washed with ice-cold saline, kept in 10 % formalin for further histopathological examination.

#### Histopathology examination

For routine H&E staining (renal and liver tissue), paraffin blocks were prepared from tissue samples, fixed in 10 % neutral buffered formalin solution after a routine tissue deparafinization process. From each tissue sample, 4 μm thick sections were obtained and stained with routine Hematoxylin-Eosin stain, and examined under light microscope. The liver activity index was scored as described by Ishak et al.[29], with the permission from authors and publisher for modification (Table 1) and according to NASH Clinical Research Network scoring system [30] as described in Table 2.

**Table 1:** Modified Histological Activity Index – grading: necro-inflammatory scores

	SCORE
**A. Periportal or periseptal interface hepatitis (piecemeal necrosis)**	
Absent	0
Mild (focal, few portal areas)	1
Mild/moderate (focal, most portal areas)	2
Moderate (continuous around < 50 % of tracts or septa)	3
Severe (continuous around > 50 % of tracts or septa)	4
**B. Confluent necrosis**	
Absent	0
Focal confluent necrosis	1
Centrolobular necrosis in some areas	2
Centrolobular necrosis in most areas	3
Centrolobular necrosis + occasional portal–central (P-C) bridging	4
Centrolobular necrosis + multiple P-C bridging	5
Panlobular or multilobular necrosis	6
**C. Focal (spotty) lytic necrosis, apoptosis, and focal inflammation**	
Absent	0
One focus or less per 10× objective	1
One to four foci per 10× objective	2
Five to 10 foci per 10× objective	3
More than 10 foci per 10× objective	4
**D. Portal inflammation**	
None	0
Mild, some or all portal areas	1
Moderate, some or all portal areas	2
Moderate/marked, all portal areas	3
Marked, all portal areas	4
**Maximum possible score for grading**	**18**

Data (adapted to new terminologies) from Ishak et al. [29]., with the permission from authors and publisher

**Table 2:** NASH Clinical Research Network scoring system definition [30]

Definition	SCORE
**A. Steatosis grade**	
< 5 %	0
5- 33 %	1
34- 66 %	2
> 66 %	3
**B. Lobular inflammation/ congestion**	
None	0
< 2	1
2–4	2
> 4	3
** C. Hepatocyte ballooning**	
None	0
Few ballooned cells	1
Many ballooned cells	2
**Maximum possible score for grading**	**8**

For sections of bone: samples were embedded in 100 ml of 5 % nitric acid, the solution was changed once in 3 days. After ensuring complete decalcification, the tissues were washed using distilled water for 30 min, following which the specimens were subjected to manual tissue processing. After processing, the tissues were embedded in paraffin and were sectioned to a thickness of 7–8 μm using the soft tissue microtome. The sections were then stained by Hematoxylin and Eosin and examined under light microscope. Image analysis was performed using a Leica Application Suite Version 4.12.0 image analysis system (Wetzlar, Germany).

Quantification of inflammatory cells was done semiquantitative using the hot spot method where the area of highest inflammatory infiltrate was chosen in each section through screening of the slide on low power examination (x100). Then non overlapped high-power fields (x400) were examined and the number of inflammatory cells was counted in each field. The mean number of inflammatory cells per high power field was calculated.

A summarization of the experimental design and methods is presented in the graphical abstract and available as Supplementary file (1).

### Statistical analysis

Values are presented as means ± SD (n = 8). Data were analyzed using one-way analysis of variance (ANOVA), followed by Tukey multiple comparison *post hoc* test. For abnormally distributed data, Mann-Whitney Test was used to analyze two independent populations. The differences were considered to be significant at *p* < 0.05. The graphs were drawn using Prism computer program (GraphPad software Inc. V5, San Diego, CA, USA).

## Results

### Effect of neostigmine with diclofenac or celecoxib on analgesia

**Fig. 1:**
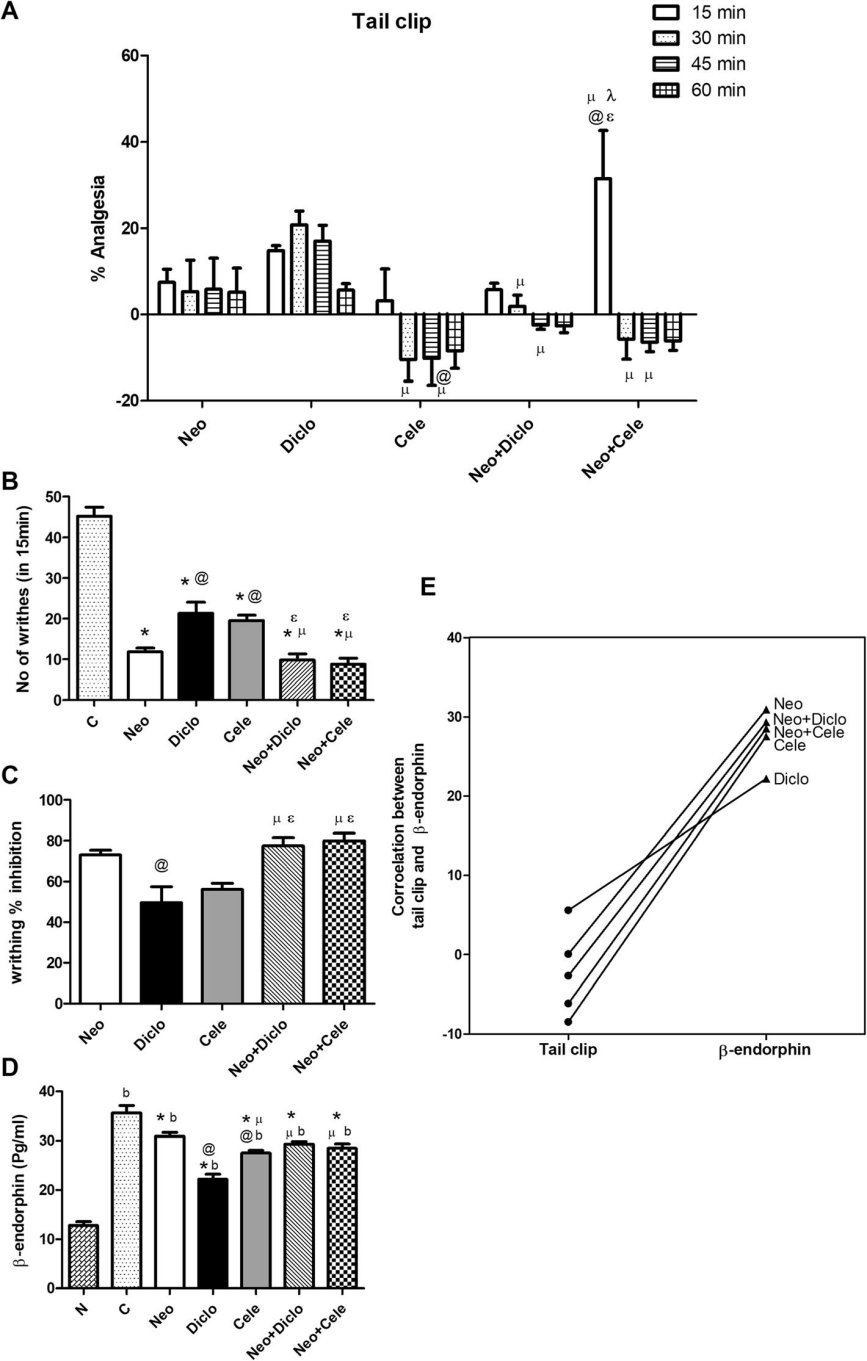
Effect of neostigmine with diclofenac or celecoxib on analgesia in rats. **a** Tail clip, **b** and **c** writhing test, **d** measuring β-endorphin serum level after 60 min of drug administration, and **e** correlation between % analgesia of tail clip with serum β-endorphin after 60 min of drug administration showing the same pattern where combination drugs show decrease in analgesic effect compared to neostigmine. Values are presented as mean ± SD. Data are compared with normal [N] (b), positive control [C] (*), neostigmine [Neo] (@), diclofenac [Diclo] (µ), celecoxib [Cele] (ɛ) and neostigmine + diclofenac [Neo + Diclo] (λ), (Mann-Whitney test for comparison) at *p* < 0.05. [Neo + Cele] neostigmine + celecoxib

As presented in Fig. 1a, the results of the tail clip show a time-dependent change in analgesia. The addition of neostigmine to celecoxib has boosted the analgesic effect of celecoxib after 15 min by 90 % compared to celecoxib group, an effect that decreased dramatically afterwards. On the other hand, addition of neostigmine to diclofenac has shown a much less analgesic activity, an effect that was not significantly different from diclofenac monotherapy. Figure 1b and c illustrate the result of the acetic acid induced writhing test in 15 min observation period. Addition of neostigmine to either diclofenac or celecoxib decreased the number of writhes significantly compared to diclofenac or celecoxib alone (i.e. 9.833 ± 2.242 vs. 21.33 ± 4.030 for diclofenac, and 8.833 ± 2.151 vs. 19.50 ± 2.078 for celecoxib). Thus, percentage of analgesia was maximum in combination of neostigmine with celecoxib (79.99 %), followed by the combination with diclofenac (77.51 %), then neostigmine alone (73.00 %).

The measurement of β-endorphin serum level after 60 min from drug application came in accordance with the results of tail clip, where all drugs have shown a definite decrease in their analgesic effect proving their short duration of action (Fig. 1d and e).

### Effect of neostigmine with diclofenac or celecoxib on acute and sub‐chronic inflammation

**Fig. 2:**
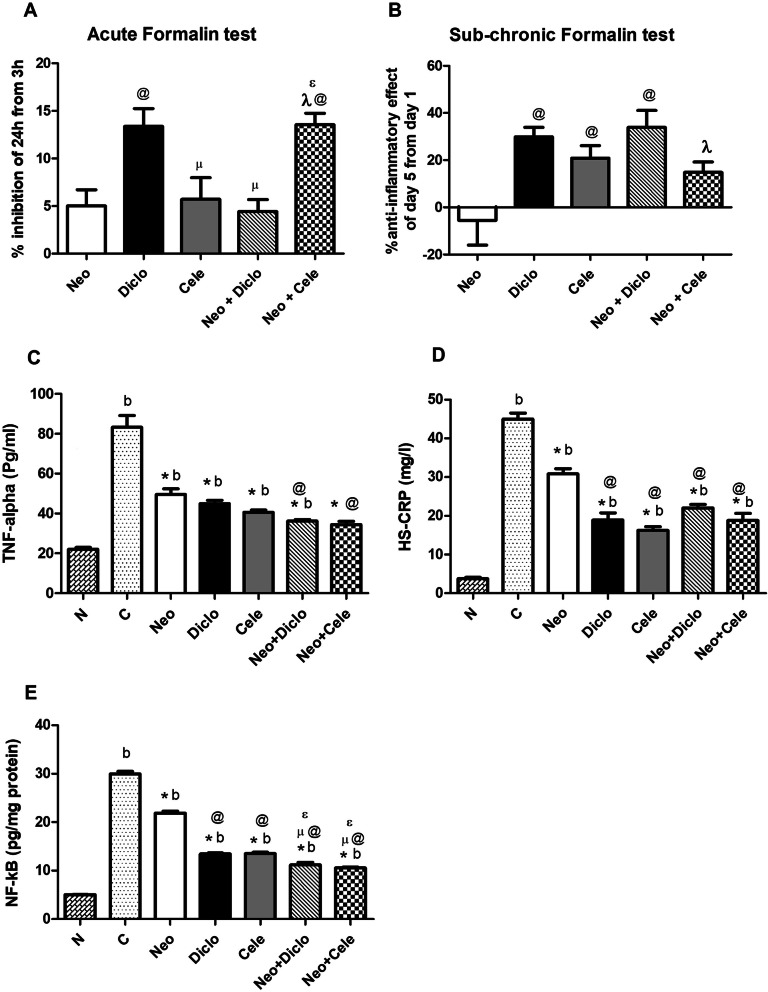
Effect of neostigmine with diclofenac or celecoxib on acute and sub-chronic inflammation. **a** Percent inhibition of inflammation in rat hind paw after 24 h of formalin injection. **b** Percent anti-inflammatory effect on rat hind paw after 5 days from formalin injection. **c** the level of TNF-ɑ, **d** HS-CRP and **e** NF-кB in serum of rats after 5 days from formalin injection in rat hind paws. Values are presented as mean ± SD. Data are compared with normal [N] (b), positive control [C] (*), neostigmine [Neo] (@), diclofenac [Diclo] (µ), celecoxib [Cele] (ɛ) and neostigmine + diclofenac [Neo + Diclo] (λ), (Mann-Whitney test for comparison) at *p* < 0.05. [Neo + Cele] neostigmine + celecoxib

The acute anti-inflammatory activity testing (Fig. 2a) shows that addition of celecoxib to neostigmine has boosted the anti-inflammatory activity of both drugs reaching a % inhibition of 13.54 % compared to neostigmine (5.02 %) and celecoxib (5.71 %). This effect was comparable to the anti-inflammatory effect of diclofenac monotherapy in 24 h. Upon sub-chronic treatment for 5 days, the neostigmine group has shown loss of anti-inflammatory activity. Addition of celecoxib to neostigmine could increase its anti-inflammatory effect, but not up to the level of the celecoxib group or diclofenac group. On the other hand, addition of diclofenac to neostigmine increases the anti-inflammatory activity of both drugs after 5 days treatment reaching (33.91 %) compared to (29.98 %) for diclofenac and (-5.51 %) for neostigmine. The results of TNF-ɑ (Fig. 2c) and HS-CRP (Fig. 2d) levels showed a statistically significant decrease in all groups compared to the positive control, where both combination therapies have shown significant increase in the anti-inflammatory effect compared to neostigmine monotherapy. Results of the NF-кB (Fig. 2e) came in accordance with TNF-ɑ and confirmed previous observations.

### Effect of neostigmine with diclofenac or celecoxib on cox activity

**Fig. 3:**
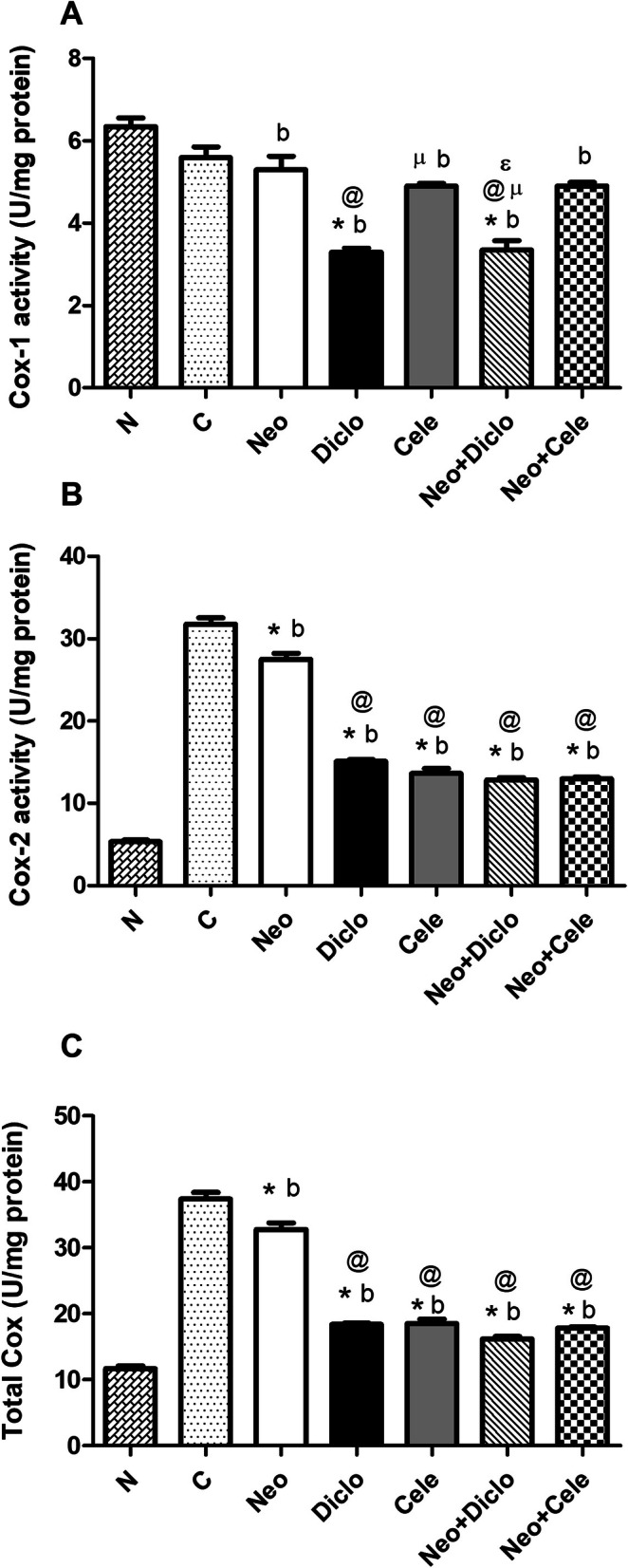
Effect of neostigmine with diclofenac or celecoxib on Cox activity after 5 days from formalin injection. **a** Cox-1 activity, **b** Cox-2 activity and **c** Total Cox activity. Values are presented as mean ± SD. Data are compared with normal [N] (b), positive control [C] (*), neostigmine [Neo] (@), diclofenac [Diclo] (µ) and celecoxib [Cele] (ɛ), (Mann-Whitney test for comparison) at *p* < 0.05. [Neo + Diclo] neostigmine + diclofenac; [Neo + Cele] neostigmine + celecoxib

In general, the specific activity of Cox-1 represented only a small fraction of total Cox activity, while Cox-2 represented most of such activity. (Fig. 3) The results of Cox-2 and total Cox activities showed a statistically significant decrease (*p* < 0.05) in all groups compared to the positive control whereas, the activity of Cox-1 showed no significant variations.

### Effect of neostigmine with diclofenac or celecoxib on kidney function

**Fig. 4:**
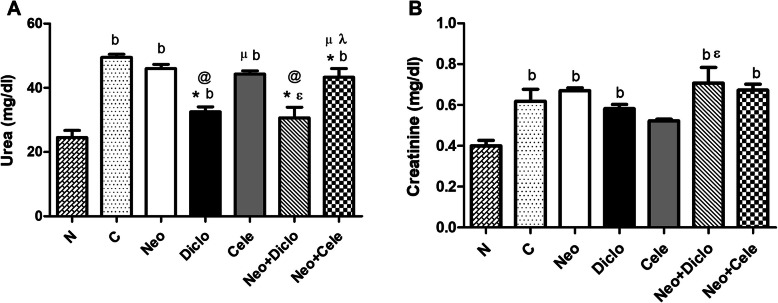
Effect of neostigmine with diclofenac or celecoxib on kidney function after 5 days from formalin injection. **a** Serum urea and **b** serum creatinine. Values are presented as mean ± SD. Data are compared with normal [N] (b), positive control [C] (*), neostigmine [Neo] (@), diclofenac [Diclo] (µ), celecoxib [Cele] (ɛ) and neostigmine + diclofenac [Neo + Diclo] (λ), (Mann-Whitney test for comparison) at *p* < 0.05. [Neo + Cele] neostigmine + celecoxib

Measurement of serum urea levels revealed a disturbed kidney function in groups treated with neostigmine and celecoxib either alone or combination reaching values of 46.00 ± 2.04 Neo, 44.25 ± 1.65 Cele and 47.00 ± 4.74 Neo + Cele *v*s 24.50 ± 3.40 N (Fig. 4a). This in turn reflects on the serum creatinine levels as shown in Fig. 4b. On another note, the combination of Neo + Diclo has shown no statistically significant difference from normal serum urea levels while it has shown disturbed serum creatinine levels.

### Effect of neostigmine with diclofenac or celecoxib on liver function

**Fig. 5:**
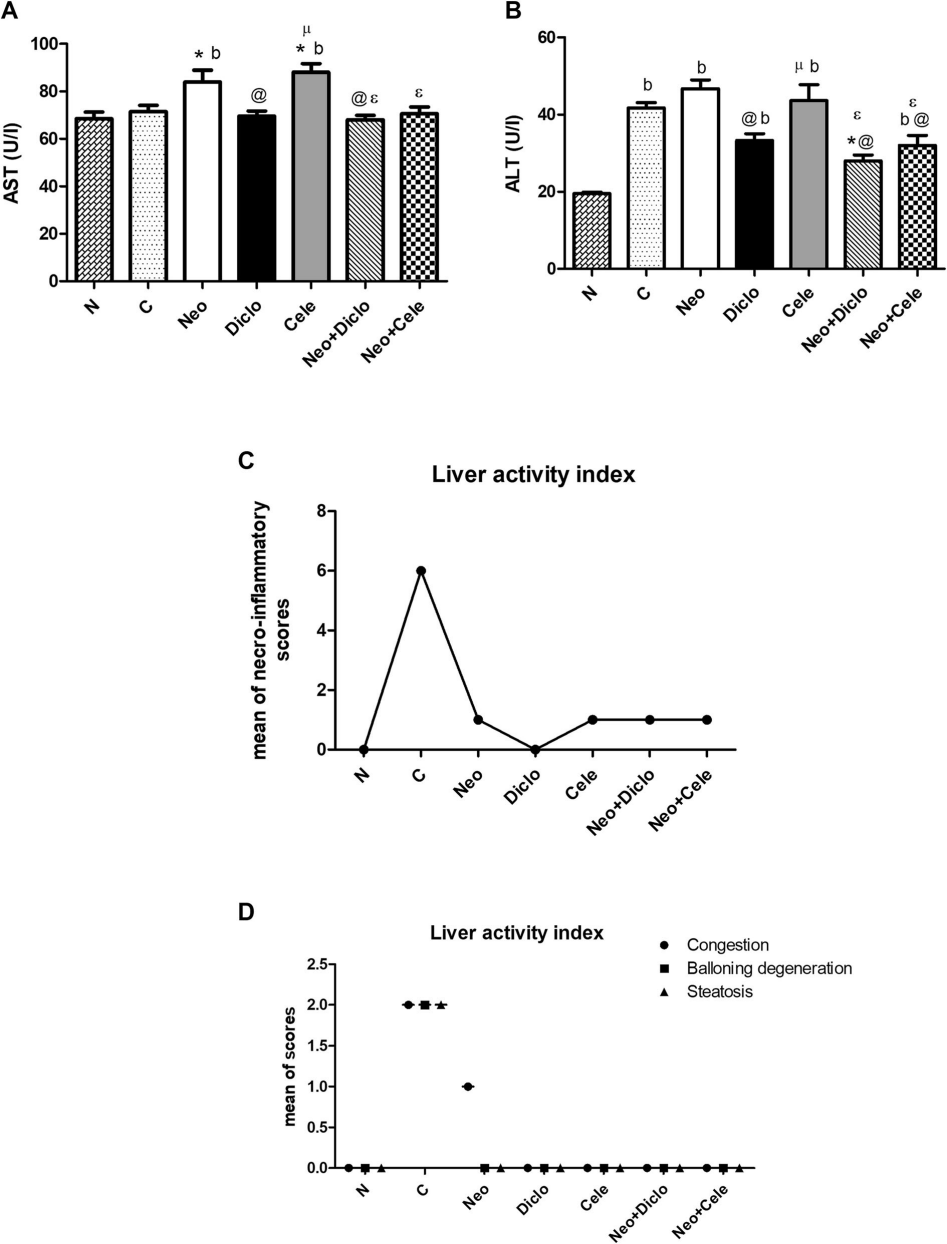
Effect of neostigmine with diclofenac or celecoxib on liver function after 5 days from formalin injection. **a** Serum AST, **b** serum ALT, **c** mean of histopathological necro- inflammatory scoring of liver using modified Ishak scoring system and **d** mean score of other histopathological liver activity indices using NASH Clinical Research Network scoring system. Values are presented as mean ± SD. Data are compared with normal [N] (b), positive control [C] (*), neostigmine [Neo] (@), diclofenac [Diclo] (µ) and celecoxib [Cele] (ɛ), (Mann-Whitney test for comparison) at *p* < 0.05. [Neo + Diclo] neostigmine + diclofenac; [Neo + Cele] neostigmine + celecoxib

The normal concentrations of AST and ALT have been determined upon serum analysis of healthy control rats presented as means of (68.50 ± 4.42 U/L and 19.50 ± 0.65 U/L) respectively. No significant elevations of AST serum level were observed in combination treated groups over the healthy control (Fig. 5a), where combination of Neo + Cele has shown significant elevation of ALT serum level compared to healthy control (Fig. 5b). Worth mentioning that the combination of diclofenac with neostigmine revealed better results than the combination with celecoxib reaching (68.00 ± 3.03 U/L vs. 76.50 ± 6.60 U/L) for AST and (28.00 ± 2.35 U/L vs. 36.50 ± 5.38 U/L) for ALT. However, no difference in effect was obvious between the different treated groups upon histopathological examination, proving the safety of both combinations as well as single drugs (Fig. 5c and d).

### Histopathological examination of hind paws from rats treated with neostigmine with/ without diclofenac or celecoxib

**Fig. 6:**
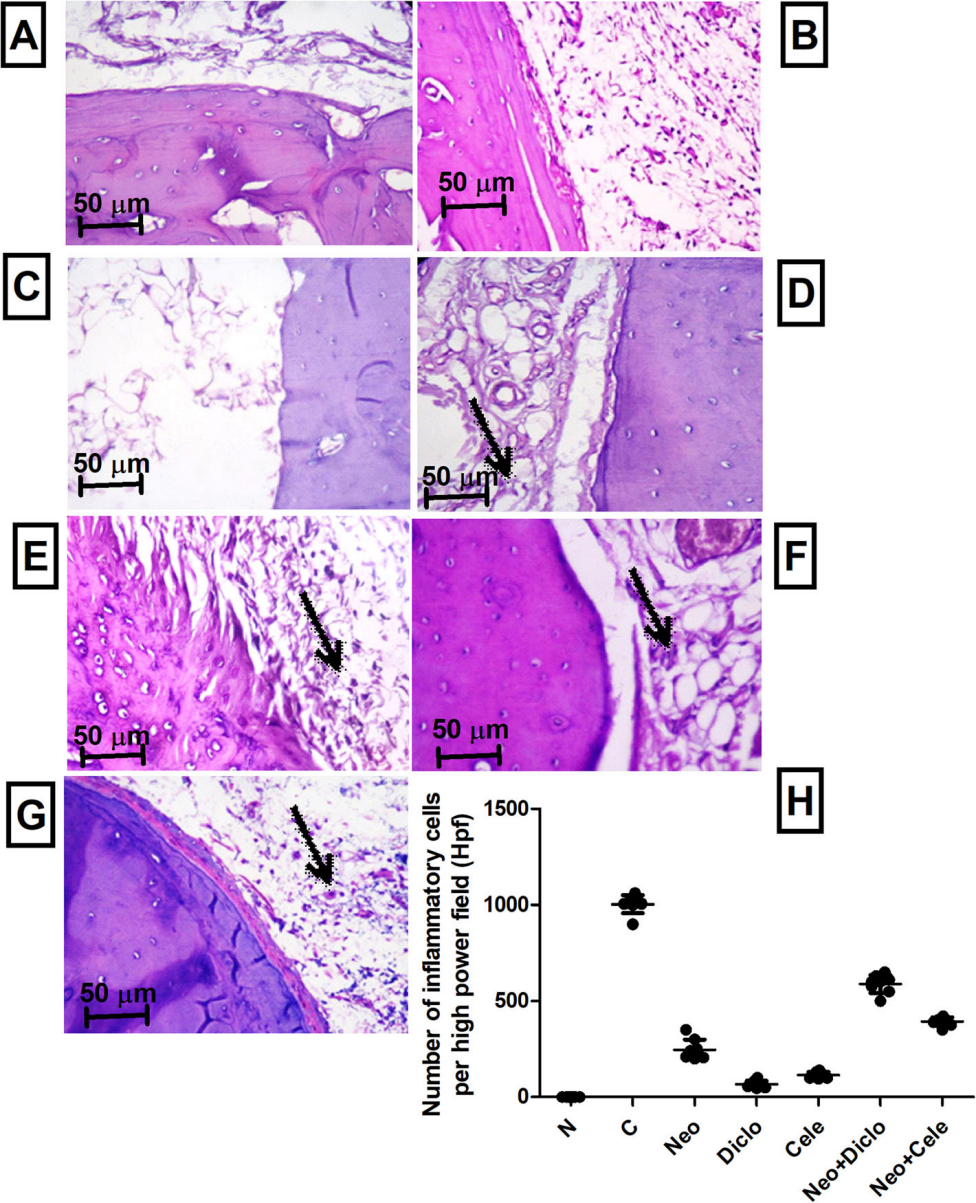
Histopathological examination of hind paws from rats treated with neostigmine with/ without diclofenac or celecoxib. **a** normal hind paw tissue showing no inflammation, **b** positive control showing marked inflammation and lympho-histocytic infiltration, **c** diclofenac treated rats with very minimal inflammation, **d** neostigmine treated rats showing moderate inflammation and lympho-histocytic infiltration (arrow), **e** neostigmine + diclofenac treated rats showing moderate inflammation and lympho-histocytic infiltration, **f** celecoxib treated rats showing mild inflammation and lympho-histocytic infiltration, **g** neostigmine + celecoxib treated rats showing moderate inflammation and lympho-histocytic infiltration, **h** number of inflammatory cells per high power field showing statistically significant difference between all different groups; except Diclo and Cele were not significantly different from each other. Values are presented as mean ± SD. Data are compared with normal [N], positive control [C], neostigmine [Neo], diclofenac [Diclo], celecoxib [Cele], neostigmine + diclofenac [Neo + Diclo], and neostigmine + celecoxib [Neo + Cele] using ANOVA, followed by Tukey multiple comparison *post hoc* test at *p* < 0.05. Low power views (x100) of the photomicrographs are present in Supplementary file (2)

As shown in Fig. 6, histopathological examination of the hind paws revealed a significant decrease in inflammation in all treated groups compared with AA rats. The best anti-inflammatory effect was observed in the diclofenac treated group, followed by celecoxib, then neostigmine. Combination of Neo + Cele has shown better effect than Neo + Diclo as depicted in Fig. 6h.

### Histopathological examination of liver and, kidney from rats treated with neostigmine with/ without diclofenac or celecoxib

**Fig. 7:**
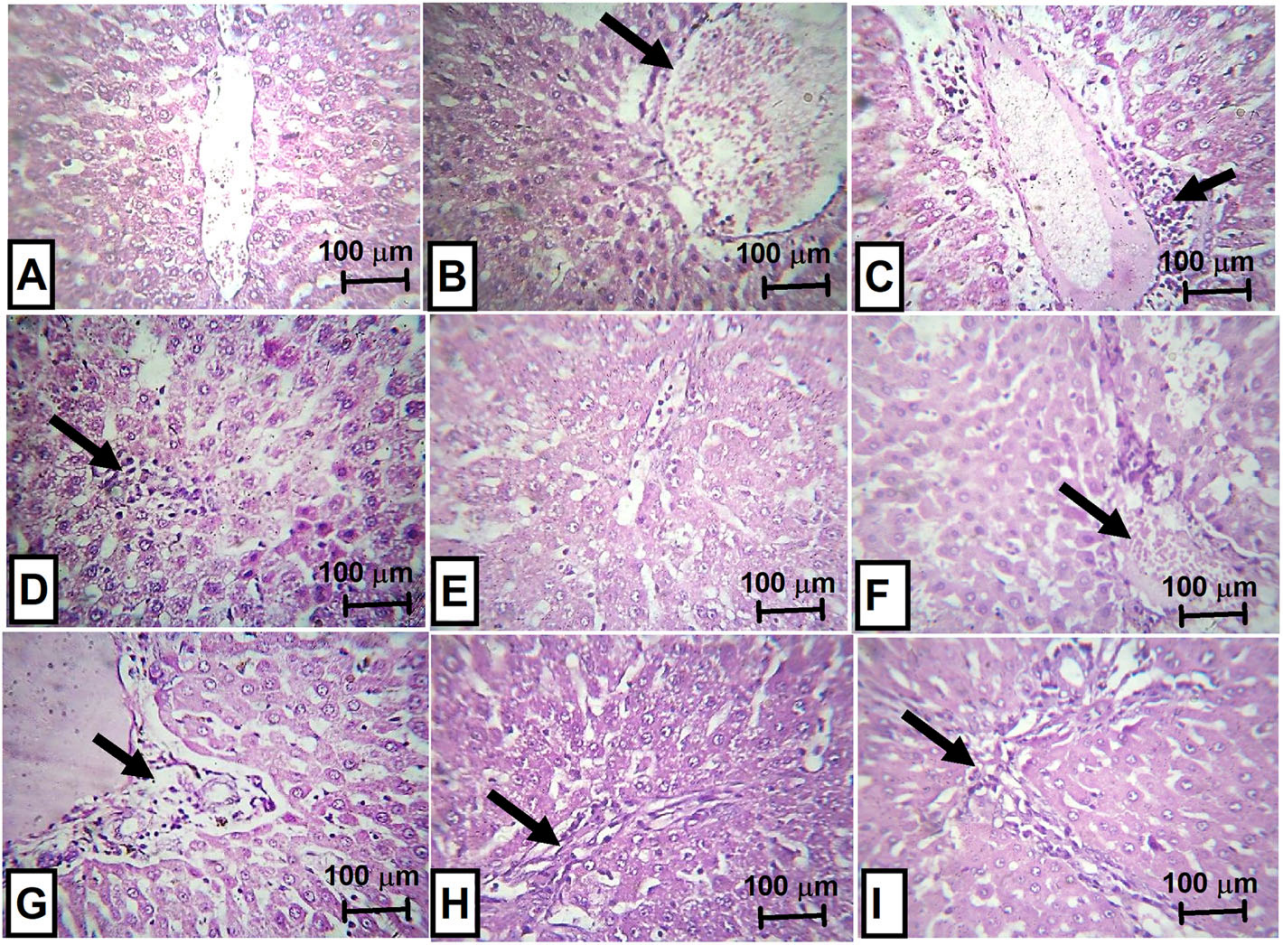
Histopathological examination of liver from rats treated with neostigmine with/ without diclofenac or celecoxib. **a** normal liver tissue, **b** positive control showing markedly ectatic congested central veins (arrow), **c** positive control showing peri-portal inflammation (arrow), **d** positive control showing focal lytic necrosis (arrow), **e** diclofenac treated rats showing normal appearance, **f** neostigmine treated rats showing mild congestion and portal inflammation (arrow), **g** neostigmine + diclofenac treated rats showing mild peri-portal inflammation (arrow), **h** celecoxib treated rats showing mild peri-portal inflammation (arrow), and **i** neostigmine + celecoxib treated rats showing mild peri-portal inflammation (arrow). Magnification power (x200)

Histopathological examination of the livers from positive control rats (Fig. 7b) revealed moderate focal lytic necrosis, portal and periportal inflammation with moderate congestion and steatosis with no detected confluent necrosis or steatosis. For the group treated with neostigmine, mild peri-portal inflammation and congestion were still noted, while other changes disappeared (Fig. 7f). Diclofenac treated group showed near normal results (Fig. 7e). For celecoxib (Fig. 7h) as well as the combination drugs (Fig. 7g and i) all showed mild peri-portal inflammation only with recovery of all other changes but with no detected histologic differences between the three groups.

**Fig. 8:**
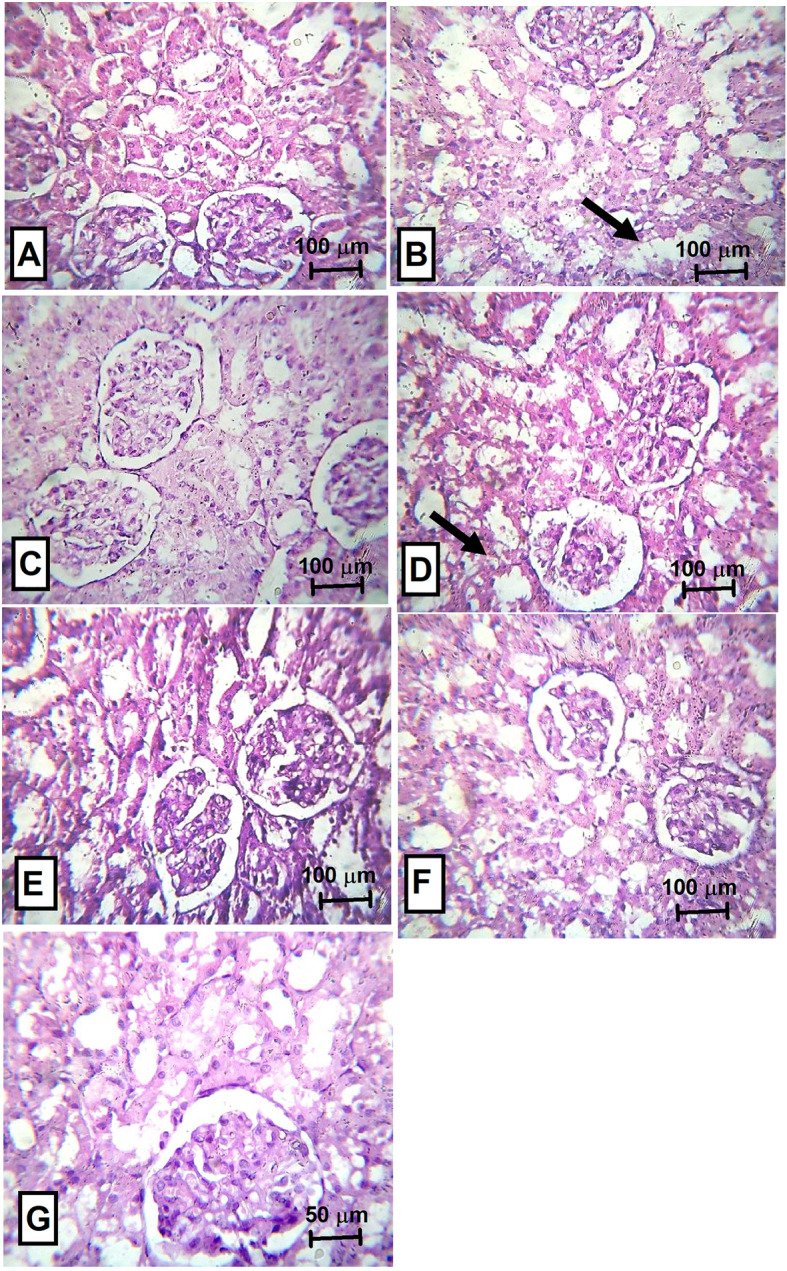
Histopathological examination of kidney from rats treated with neostigmine with/ without diclofenac or celecoxib. **a** normal renal tissue, **b** positive control showing moderate cloudy swelling of the tubules (arrow), **c** diclofenac treated rats showing normal appearance of the tubules and glomeruli, **d** neostigmine treated rats showing mild cloudy swelling (arrow), **e** neostigmine + diclofenac treated rats showing normal appearance, **f** neostigmine + celecoxib treated rats showing normal appearance and **g** celecoxib treated rats showing near normal renal tissue. Magnification power for A-F (x100) and for G (x400)

Examination of the kidneys revealed for the positive control moderate cloudy swelling of the renal tubules with unremarkable glomeruli (Fig. 8b). Neostigmine monotherapy has shown mild cloudy swelling (Fig. 8d). For all other groups near normal kidney sections are retrieved.

## Discussion

This study focused on the combination of neostigmine with the non-selective Cox inhibitor, diclofenac, or the selective Cox-2 inhibitor, celecoxib. This rationale was based on the facts that the anti-nociceptive effect of NSAIDs is Cox mediated as stated by Miranda et al. [4] On the other hand, neostigmine, being an acetylcholinesterase inhibitor, should possess anti-inflammatory properties as seen in several studies examining the cholinergic anti-inflammatory pathway [9, 11].

Comparing both combinations in the tail clip test of this study, Neo + Cele reveals a boosting onset of analgesic action within 15 min, but short duration, fading after less than 30 min. In contrast, Neo + Diclo reveals rapid onset with lesser magnitude and decrease in analgesia right after 30 min. Measuring β-endorphin serum level after 60 min has proven the loss of analgesic activity of all treated groups, while writhing tests has proven the effective analgesia of both combinations within 15 min period. This outcome proves previous observation by Miranda et al. [4] that selective Cox-inhibition is not yet enough to identify the strong analgesic property of NSAIDs. Many others studies have shown that celecoxib has less analgesic effect than other NSAIDs and that its effect depends on the duration of administration, where it is preferred for short-term treatment periods due to reversibility of its effect [31–33].

The fact that Neo + Diclo in this study didn’t show any additive or synergistic analgesic effect compared to Neo alone, came in contradiction to the study by Miranda et al.[34] who proved a synergistic analgesic effect of combining neostigmine with diclofenac, attributing this effect to the increased acetylcholine concentration in the synaptic cleft, which in turn gives a signal of supraspinal antinociception. Yet, adding celecoxib to neostigmine caused a clear, instant synergism in the tail clip, a result that needs more investigation and could be of important clinical implications for rapid manipulation of acute pain states.

Formalin injection is a well-known model of persistent inflammatory pain in experimental animals, which is characterized by edema and inflammation. The release of histamine, bradykinin, serotonin, substance P and prostaglandins induce the inflammatory reaction. These chemical substances cause an increase in vascular permeability, which promote the accumulation of fluid in interstitial tissue [35]. Injection of the rat paw with formalin causes tissue damage and evokes a biphasic spinal release of prostaglandin E2 that increases in concentration of 110 % in the 0–10 min period and then 83 % in the 20–30 min period after formalin injection, with no further increase after 30–60 min of formalin injection [36]. It is well known, that NSAIDs reduce the nociceptive behavior during the second phase (20–30 min), while the first phase is not affected [35].

Accordingly, in this study, the anti-inflammatory properties of neostigmine have shown a clear synergistic effect within 24 h when added to celecoxib (Neo + Cele), which then declined after 5 days of the study, an effect that is attributed to Neo rather than Cele. This outcome comes in accordance with the study of Nishyama [37], who has shown the same effect upon studying celecoxib in acute formalin test. Knowing that in the design of the acute anti-inflammatory test, the drugs have been administered before the challenge with formalin, would encourage the clinical injection of the combination (Neo + Cele) before the onset of inflammation. Surprisingly, the Neo + Diclo combination had an opposite outcome, as it didn’t show a significant effect after 24 h, but caused clear potentiation after 5 days. However, analyzing serum samples after 5 days, both combinations Neo + Cele and Neo + Diclo have shown an increase in the anti-inflammatory properties of neostigmine as seen in the results of both TNF-α, NF-кB and HS-CRP.

Histopathological examination of the inflamed hind paws after 5 days came in accordance with the biochemical data, where all drugs have shown decreased inflammation. Diclofenac and celecoxib monotherapy have shown much better effect than neostigmine. However, combination therapy has shown delayed histopathological improvement compared to neostigmine monotherapy. This discrepancy from the biochemical data is attributed to the short treatment protocol of this study (5 days), where histopathological improvements require chronic treatment [38]. Moreover, a study by Álvarez-Soria et al. [39] matched the outcome of our combination therapy, where treatment with celecoxib and classic NSAID improved joint pain and function, decreased Cox-2 expression, but synovial macrophage infiltration were less affected. As specified by Tsuboi et al. [40] inflammatory cell infiltration and vascularization continues even with improved clinical outcome of the joint in RA patients, indicating a subclinical inflammation.

The positive feedback loop of the Cox-2-PGE_2_-EP_2_-NF-κB signaling pathway is well known to initiate and preserve inflammation [41]. Activated Cox-2 increase the production of PGE_2_, that binds to the EP_2_ receptor and consequently activates NF-κB; the important inducer of pro-inflammatory cytokine [41, 42]. More than twenty-five cytokines are controlled directly by NF-κB. On the other hand, high levels of TNF-α promptly activates Cox-2 and NF-κB in a positive feedback loop [43]. In a study of Walker et al. [44] celecoxib has shown a similar Cox-2 selectivity ratio to diclofenac. In contrast, comparing in vivo findings of both drugs, same therapeutic concentrations have shown different effect on Cox-1. Accordingly, in our study, diclofenac (monotherapy and in combination with Neo) has shown more decrease in Cox-1 activity than celecoxib. Both drugs, however, had same effect on Cox-2 activity.

Expanding to the previous findings, a mouse model of Saccular Intracranial Aneurysms has shown that endothelia cells-activated NF-κB activity was suppressed with the administration of the selective Cox-2 inhibitor celecoxib [41]. Moreover, the results of the Cox isoforms activity study in the present work come in accordance with a previous work from Nishiyama et al. [37], where intrathecal administration of celecoxib resulted in a dose dependent inhibition of the flinch response of the formalin test, but had no significant effect on the tail flick latency. With doses up to 200 mg, no hemodynamic changes or behavioral side-effects were observed [37].

Clinically, neostigmine is being used for its analgesic effect in both the perioperative and postoperative phases with different patients proving high efficacy [45, 46]. It is well documented, that any surgical procedure causes perioperative immunosuppression and neuroendocrine stress, exerted by activation of the autonomic nervous system and the hypothalamic-pituitary-adrenal axis [47]. Moreover, intraoperative blood pressure management, blood transfusion, hyperglycemia, hypothermia and the anesthetic agent itself cause also perioperative immunosuppression, which persist for at least few days post operation [48].

Numerous studies demonstrated that commonly used anesthetic agents in surgery and intensive care units possess anti-inflammatory properties and could impair the inflammatory response process either directly or indirectly by modulating the stress response [49, 50]. However, anti-inflammatory properties of anesthetic agents is therapeutically beneficial in only very distinct situations, where it’s immunosuppressive properties affect the long term outcomes of most patients after surgery [51, 52].

According to the current study, adding a combination of Neo + Diclo or Neo + Cele to the anesthetic agent in the perioperative phase might promote the anti-inflammatory characteristics of neostigmine adding to the anti-inflammatory effect of the anaesthetic agent. The clinical implication of such combinations in anesthesia requires more in vivo studies.

The nephrotoxicity of the conventional NSAIDs as well as selective Cox-2 inhibitors is well known, where both Cox isoforms play a crucial role in maintaining renal function homeostasis [53]. Accordingly, all treated groups of this study seemed to disturb some kidney functions as seen in the results of serum urea and creatinine, however with normal histopathological appearance of the kidney, which may relate to the low dose of diclofenac and celecoxib used in this study. This comes in accordance with Izhar et al. who claims that the side effects seen by diclofenac and celecoxib are related to their dosing frequency rather than their sensitivity [54].

The effect of Diclo on the liver in the current study has shown, surprisingly, complete safety as reflected on the serum AST, serum ALT and histopathological examination results. Known that diclofenac is usually categorized of being high toxic [55], the normal appearance in this study might be related to the low dose used.

Neostigmine as well as celecoxib appears to disturb liver functions moderately as seen in the serum AST/ALT results and the histopathological pictures. However, combining both drugs has shown to be safer than each drug alone. The hepatotoxicity of celecoxib within therapeutic doses has been intensively studied earlier and it was recommended to be used with caution in patients with liver insufficiency [56].

## Conclusions

In conclusion, adding neostigmine to diclofenac or celecoxib causes an increased level of analgesia of both NSAIDs with a rapid onset of action and short duration (up to 15 min), while both NSAIDs could potentiate the anti-inflammatory effect of neostigmine. Hence, adding selective or non-selective NSAIDs, as examined in this study, could be a good adjunct option to general anesthetic agents and neostigmine in perioperative stage, an outcome that needs further clinical investigation.

## Supplementary Information

**Additional file 1: Supplementary File 1.** Graphical abstract, summarizing the experimental design and the methods.

**Additional file 2: Supplementary File 2.** Histopathological examination of hind paws from rats treated with neostigmine with/ without diclofenac or celecoxib in low power views (x100).

## Data Availability

All data generated or analysed during this study are included in this published article.

## Abbreviations

ACHEI: Acetylcholinesterase inhibitor
AST: Aspartate transaminase
ALT: Alanine transaminase
BL: Basal latency or control latency
Cox: Cyclooxygenase
Cele: Celecoxib
Diclo: Diclofenac
HS-CRP: High sensitivity C-reactive protein
MPE: Max possible effect
ML: Max latency or cut off time
Neo: Neostigmine
NF-кB: Nuclear Factor-кB
NSAIDs: Non-steroidal anti-inflammatory drugs
TNF-ɑ: Tumor necrosis factor- ɑ
TL: Test latency
Wc: No. of writhes in control group
Wt: No. of writhes in test group
