# Supplementary figures and images for: The effective interplay of (non-) selective NSAIDs with neostigmine in animal models of analgesia and inflammation
Source: BMC Pharmacol Toxicol. 2021 May 1;22:24. doi: 10.1186/s40360-021-00488-9 (PMC8088641; doi:10.1186/s40360-021-00488-9)

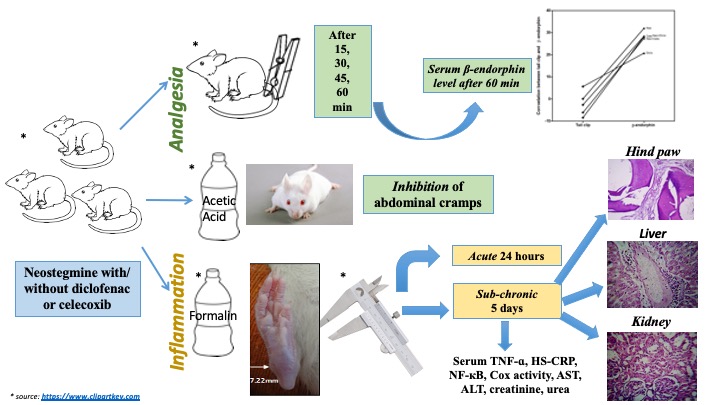

Supplement: Supplementary file 1 — Additional file 1: Supplementary File 1. Graphical abstract, summarizing the experimental design and the methods. [file 40360_2021_488_MOESM1_ESM.jpeg]

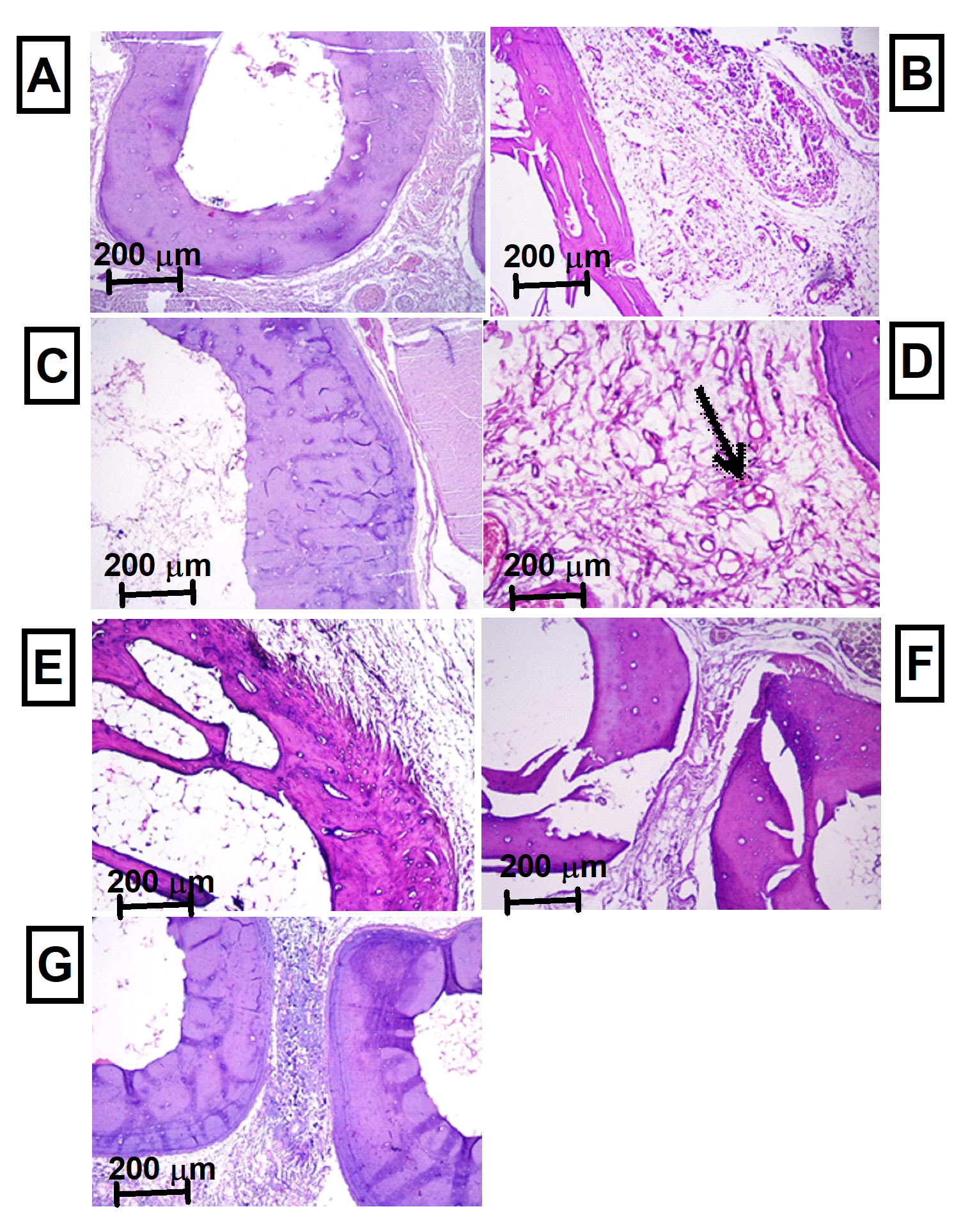

Supplement: Supplementary file 2 — Additional file 2: Supplementary File 2. Histopathological examination of hind paws from rats treated with neostigmine with/ without diclofenac or celecoxib in low power views (x100). [file 40360_2021_488_MOESM2_ESM.jpg]
